# Bony Lesions in Paediatric Acute Leukaemia

**DOI:** 10.5334/jbsr.2474

**Published:** 2021-05-20

**Authors:** Thiebault Saveyn, Nele Herregods

**Affiliations:** 1 UZ Gent, BE

**Keywords:** Pediatric radiology, Acute leukemia, Bone lesions leukemia, Metaphyseal bands, Pathologic fracture, Bone tumor

## Abstract

**Teaching Point:** Translucent metaphyseal lines in children warrant further analysis to rule out malignancy.

## Case Study

**Figure 1:**
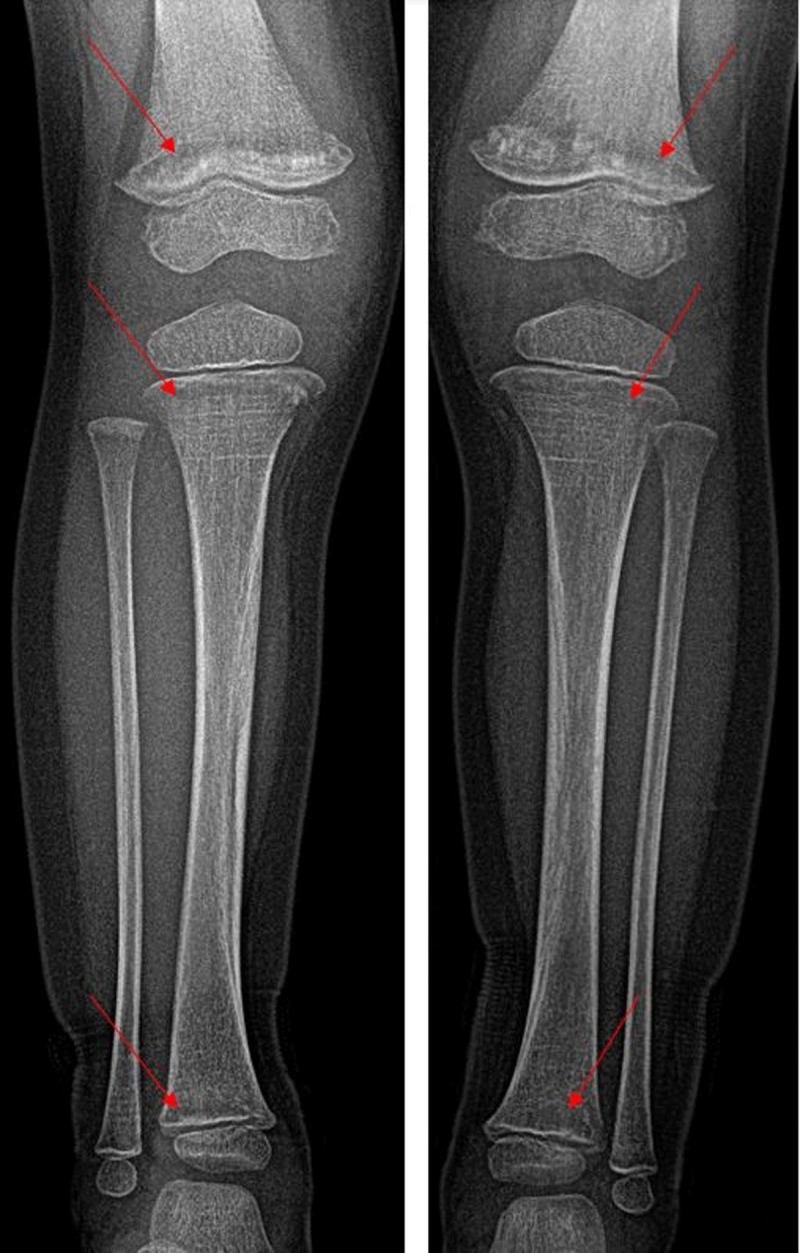


**Figure 2:**
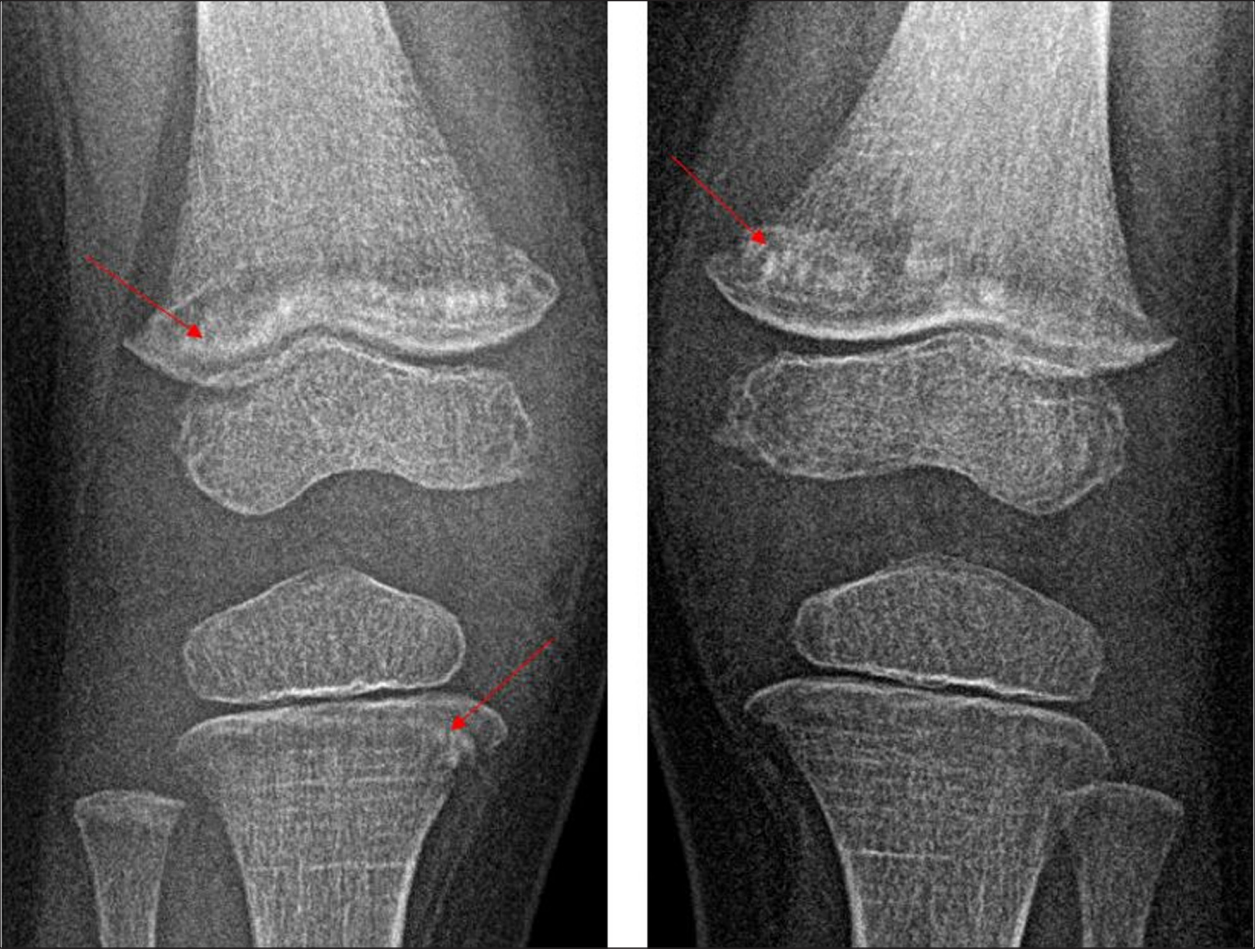


A three-year old girl was unable to walk due to aggravating pain in the limbs for three weeks. The pain began after a fall on the knee three weeks earlier. She even refused to stand up or sometimes even sit straight up. The pain improved with the intake of ibuprofen. There was no history of recent infection or fever. During clinical examination, flexion of the knee was very painful on both sides. Ultrasound didnt show any signs of synovitis or knee joint effusion. Radiographic images showed bilateral translucent metaphyseal bands in the femur and tibia (***Figure 1***). There was a (pathological) fracture of the distal metaphysis of both femora and the proximal metaphysis of the right tibia (***Figure 2***).

**Figure 3:**
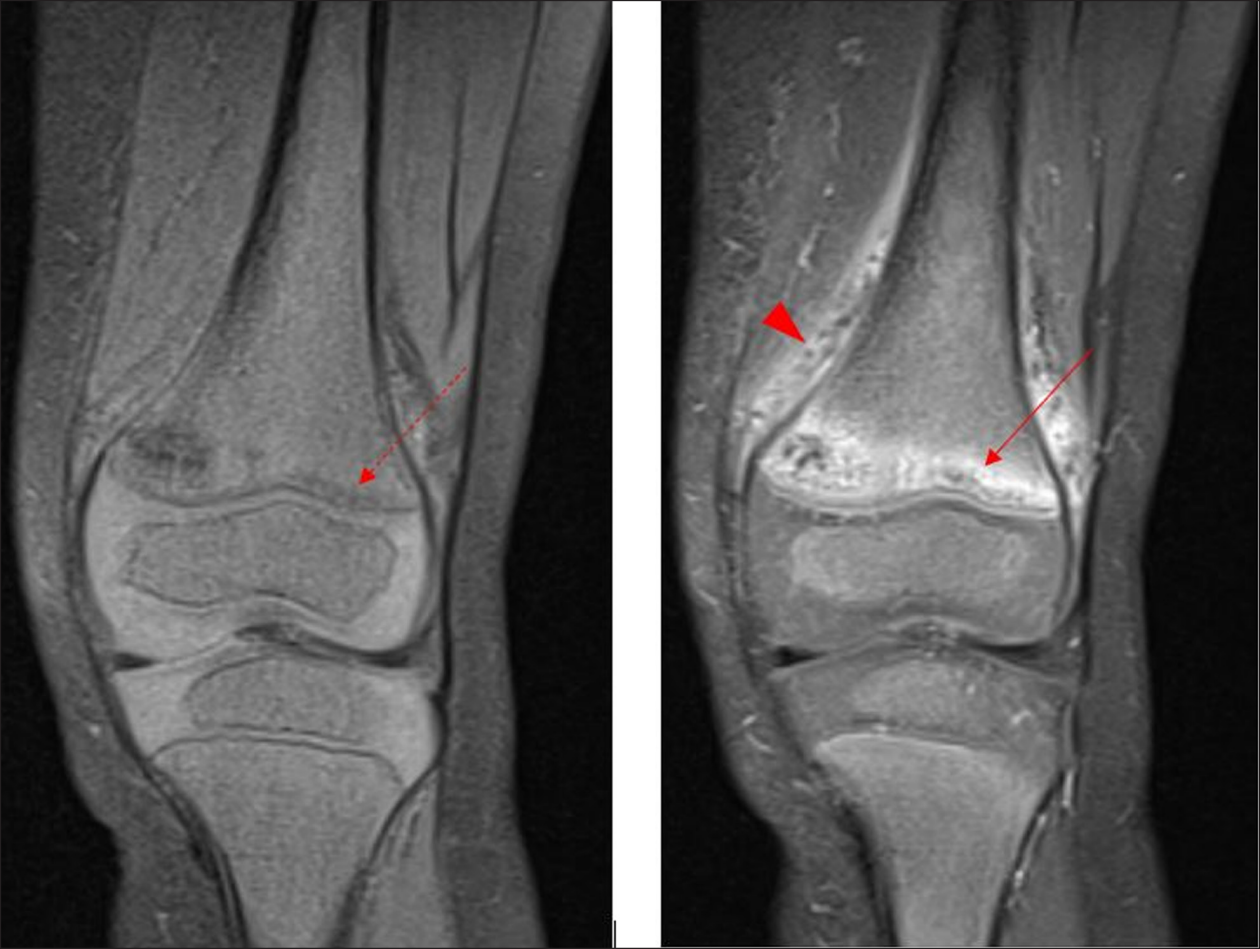


Further investigation with magnetic resonance imaging (***Figure 3***) was done and revealed high T2-signal at the level of the metaphyseal bands (arrow) with adjacent soft tissue oedema (***Figure 3***, arrowhead). The pathological fractures were also visible as T1 hypo-intense lines (dotted arrow).

A blood sample was taken and showed leukocytosis (10.000 billion/l) consisting of 8% normoblasts. This finding, along with the imaging, was enough to identify the underlying pathology as acute leukaemia.

## Comment

Acute leukaemia is a generalized myeloproliferative disorder, the most common malignancy in childhood. It often manifests as a musculoskeletal disorder. Bone pain is frequent due to massive proliferation of hematopoietic tissue, mainly in long bones and vertebral bodies. Osteoporosis and compression fractures are the main factors involved in the genesis of pain. An incidence of 41% to 75% of radiographic bony changes in children with acute leukaemia is reported [1]. Osteolytic lesions are the most frequent radiographic features, resulting from leukemic infiltration of the bone marrow, local haemorrhage, and osteonecrosis of adjacent bone. These lesions are typically found in the metaphysis of the long bones as radiolucent metaphyseal bands (previously called leukemic lines). They may nevertheless occur in flat or small bones and might be associated with periosteal reaction.

Other manifestations are vertebral collapse or fractures and osteosclerotic lesions, derived from reactive new bone formation secondary to leukemic cell infiltration and osseous infarction.
